# The relative area score for sublingual varices reliability measurement: a diagnostic study

**DOI:** 10.1186/s12903-023-03068-x

**Published:** 2023-06-06

**Authors:** Christian R. Klein, David Stoppenbrink, Jannik Geier, Andreas Mayr, Helmut Stark

**Affiliations:** 1 Department of Prosthetic Dentistry, Preclinical Education and Materials Science, Dental School, Rheinische Friedrich-Wilhelms University, University of Bonn, Welschnonnenstr. 17, 53111 Bonn, Germany; 2 Department of Oral, Maxillofacial and Plastic Surgery, Center of Dento-Maxillo-Facial Medicine, University Hospital Bonn, Welschnonnenstr. 17, 53111 Bonn, Germany; 3 Department of Medical Biometry, Informatics and Epidemiology, University Hospital Bonn, Venusberg-Campus 1, 53127 Bonn, Germany

**Keywords:** Sublingual varices, Reliability, Clinical inspection, Aging, Oral science, Maximum correlation, Clinical markers, Preventive health care

## Abstract

**Background:**

Sublingual varices (SV) and their predictive potential for other clinical parameters is a much studied topic in oral medicine. SVs have been well studied as predictive markers for many common diseases such as arterial hypertension, cardiovascular disease, smoking, type 2 diabetes mellitus and age. Despite many prevalence studies, it is still unclear how the reliability of SV inspection affects its predictive power. The aim of this study was to quantify the inspection reliability of SV.

**Methods:**

In a diagnostic study, the clinical inspection of 78 patients by 23 clinicians was examined for the diagnosis of SV. Digital images of the underside of the tongue were taken from each patient. The physicians were then asked to rate them for the presence of sublingual varices (0/1) in an online inspection experiment. Statistical analysis for inter-item and inter-rater reliability was performed in a τ-equivalent measurement model with Cronbach's $\alpha$ and Fleiss κ.

**Results:**

The interrater reliability for sublingual varices was relatively low with κ = 0.397. The internal consistency of image findings for SV was relatively high with α≈ 0.937. This shows that although SV inspection is possible in principle, it has a low reliability R. This means that the inspection finding (0/1) of individual images often cannot be reproduced stably. Therefore, SV inspection is a difficult task of clinical investigation. The reliability R of SV inspection also limits the maximum linear correlation ${r}_{max}$ of SV with an arbitrary other parameter Y. The reliability of SV inspection *R* = 0.847 limits the maximum correlation to ${r}_{max}$ (SV, Y) = 0,920—a 100% correlation was a priori not achievable in our sample. To overcome the problem of low reliability in SV inspection, we propose the RA (relative area) score as a continuous classification system for SV, which normalises the area of visible sublingual veins to the square of the length of the tongue, providing a dimensionless measure of SV.

**Conclusions:**

The reliability of the SV inspection is relatively low. This limits the maximum possible correlation of SV with other (clinical) parameters. SV inspection reliability is an important indicator for the quality of SV as a predictive marker. This should be taken into account when interpreting previous studies on SV and has implications for future studies. The RA score could help to objectify the SV examination and thus increase its reliability.

## Background

**Fig. 1:**
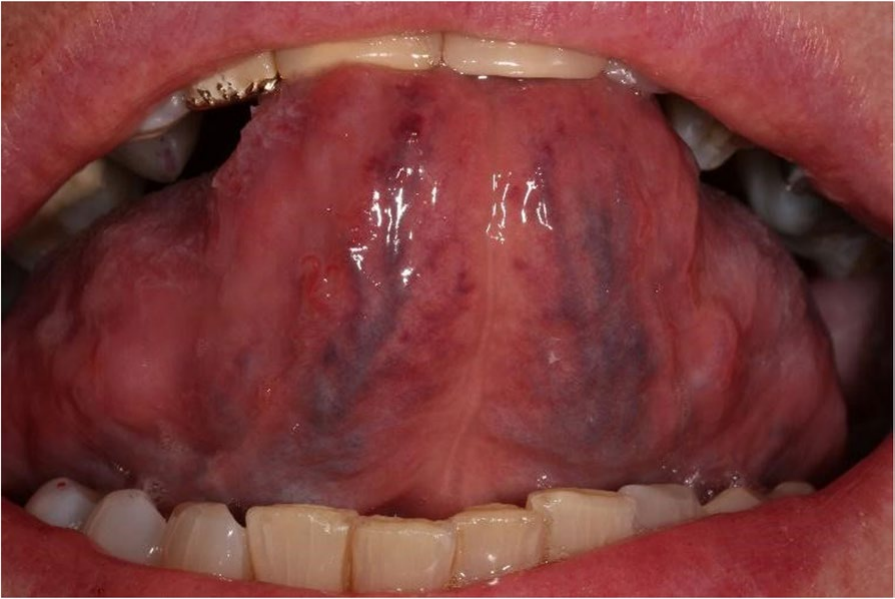
Example of SV: Dilated changes in the sublingual veins (*Vv. linguae profundae)*. Written consent obtained from patient

Sublingual varices (SV) are a widely studied topic in oral medicine. SV are dilated changes in the sublingual veins (Vv. linguae profundae, Fig. 1). Previous studies have shown a predictive value of SV for a number of clinical parameters such as arterial hypertension [1–3], smoking [4–7], type 2 diabetes mellitus [6, 8], dyslipidaemia [6, 8], thrombosis [9, 10], cirrhosis [11], allergies [12] and age [13], where SV remain persistent with increasing age [14].

The diagnosis of SV on clinical examination depends on the examiner. Despite many studies on SV, the influence of the examiner on the diagnosis of SV and consequently the accuracy of SV measurement is not clear. Previous studies of SV have been conducted on large patient samples (n > 400, dental patients) [1, 2, 5, 6, 8, 14, 15], often with an inclusion criterion of age > 40 years [1, 4, 6, 8, 14]. Many previous studies on SV used a binary 0/1 classification [1, 2, 4–6, 8, 14] by relatively few (1–2) medical experts [1, 2, 5, 6]. There is currently no objective gold standard for the diagnosis of SV on clinical inspection. This study was the first to systematically investigate the influence of the examiner on the diagnosis of SV during oral inspection. This was used to statistically quantify the influence of examiner reliability on SV quality as an indicator of other clinical parameters. In addition, the RA (relative area) score is proposed as a continuous classification system for SV to increase the reliability and objectivity of SV inspection.

## Methods

### Design and setting

Our study design to investigate the reliability of SV-inspection consists of two parts:

1. **Clinical study:** A clinical study was conducted between 2019 and 2020 in the Department of Prosthetic Dentistry, Preclinical Education and Materials Science, Faculty of Dentistry, Rheinische Friedrich-Wilhelms-Universität, University Hospital Bonn, Bonn, Germany. Patients attending the dental clinic for a routine examination or treatment were invited to participate in the study. There were no exclusion criteria regarding age or previous diseases. Prior to dental treatment, several photographs of the underside of the tongue were taken with a special camera for oral cavity photographs (measuring device: Canon EOS 800D, Canon EF 100 mm macro, Nissin MF18 macro ring flash). A total of *n* = 78 patients (43 women and 35 men) aged 22 to 82 years, were included.
2. **Experiment on clinical inspection:** The recorded images of the clinical study were presented in an arbitrary order and then rated in by m = 23 medical professionals for the detection of SV. Approximately half of the group of medical experts were medical doctors or students in clinical training, and the other half were dentists. All recruited medical experts regularly perform clinical inspections of the oral cavity in their profession or are familiar with it as part of their studies. All recruited medical experts were instructed in advance about SV as a clinical finding on examination by means of image examples.

**Fig. 2:**
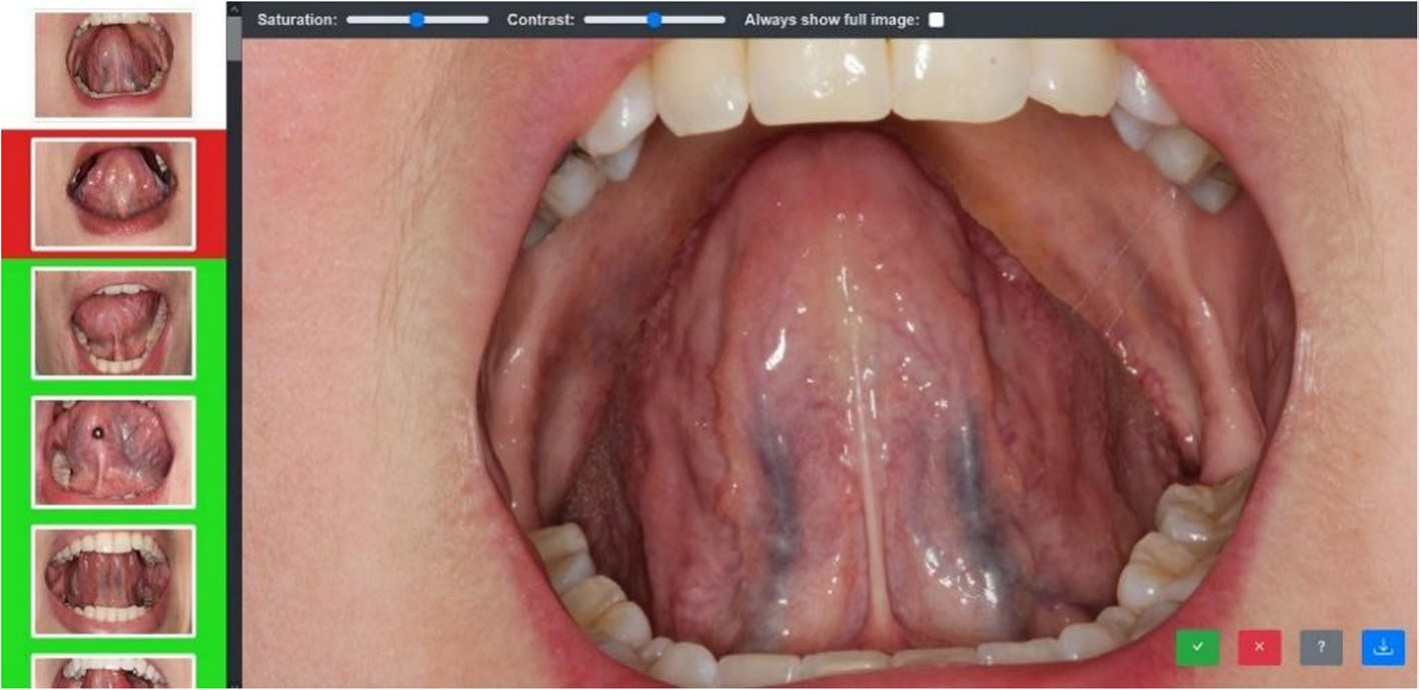
Exemplary representation of the experiment for the clinical inspection of SV. Written consent obtained from the patients. The yesnomabye programme was used for this purpose. The medical professionals were able to evaluate whether they thought SV were present or not with a simple click of the mouse. The binary findings of the individual images (1 = SV detected, 0 = no SV detected) were summarised as a vector at the end, and the data of all medical professionals were then written into a common matrix

**Fig. 3:**
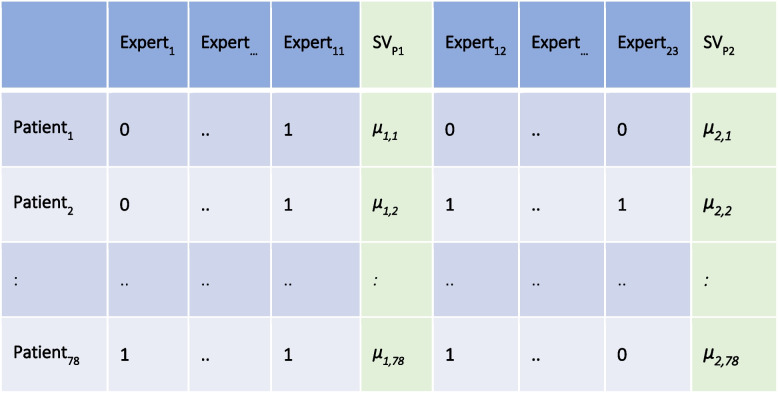
Schematic representation of the test–retest correlation in the parallel measurement model for estimating the reliability of SV inspection. The group of medical experts (m = 23) was divided into two halves and SV_P_ values were calculated for each image for both groups. Subsequently, the μ_ij_ were correlated with each other to estimate the reliability of inspection (test–retest correlation). The numbers (0,1) inserted in the matrix are used to illustrate the method and are not real measurements from the study

This experiment was conducted with a freely available computer programme called yesnomabye (figure 2, https://github.com/richard-vock/yesnomaybe). The findings of an individual expert were stored in binary form (1 = SV detected, 0 = no SV detected) and the entire inspection was stored in vector form. Subsequently, the data from all medical professionals were collated into in a matrix, a schematic illustration of the procedure is shown in figure 3.

### SVP as a surrogate parameter for SV

As there is no practical definition of SV exists yet due to the lack of a gold standard, we used the mean values of the 0/1 ratings with respect to an image of the underside of the tongue as surrogate parameter. We refer to this as SV subject prevalence (SV_P_).

### Statistical analysis

Statistical analysis was performed with Excel and ROC analysis using R. The colouring and measurement of the area A and length L used in the RA score were performed using GIMP (GNU Image Manipulation Program). The measurement of area A and length L was performed by a physician with experience in oral medicine and clinical SV inspection (one of the authors). A and L were measured twice with an interval of several days.

#### Quantification of SV inspection difficulty

In the absence of a gold standard for the analysis of SV inspection the reliability of the test scale, together with the interrater reliability, can thus serve as a measure of the difficulty of the task. A disagreement between the scores of several medical experts regarding an item is taken as an indication of the difficulty of the findings.

Since an at least essentially τ-equivalent measurement model [16] is given for the evaluation of the individual pictures (test items),we analysed the reliability of the scale with Cronbach's $\alpha :=\frac{n}{n-1}\left(1-\frac{{\sum }_{i=1}^{n}{\sigma }_{{U}_{i}}^{2}}{{\sigma }_{V}^{2}}\right)$[17].

${U}_{i} \in \{\mathrm{0,1}\}$ here is the SV rating of the clinical examination of patient $i\in \{1,\dots ,n\}$ with $n = 72$.${\sigma }_{V}^{2}$ is the variance of the frequency of SV assessment ${V}_{j}$ across all medical experts $j \in \{1,\dots ,m \}\ \mathrm{ with }\ m = 23$. ${V}_{j}:={\sum }_{\mathrm{i}=1}^{\mathrm{n}}{\updelta }_{\mathrm{ij}} \in \{1,\dots ,n\}$ counts the absolute frequency of SV assessment by medical expert $j$ over all $i = 1,...,n$ patients, where the SV assessment ${\updelta }_{i\mathrm{j}}$ of medical expert $j$ regarding patient $i$ is defined by

$${\updelta }_{i\mathrm{j}} :=\left\{\begin{array}{l}1,\, if\ medical\ expert\ j\ detects\ SV\ in\ patient\ i:{ U}_{i} = 1\\ 0,\, else\end{array}\right.$$

We quantified the interrater reliability, given multiple medians and nominal data (0/1), with Fleiss $\kappa := \frac{{p}_{\lambda }-{p}_{e}}{1-{p}_{e}}$ [18]. $\kappa$ is the proportion of the actually achieved over-random agreement ${p}_{\lambda }-{p}_{e}$ to the in principle achievable over-random agreement $1-{p}_{e}$ of the medical experts in their SV diagnosis. Let ${n}_{i1}$ be the number of medical experts who diagnosed SV in patient i and ${n}_{i0}$ the number of medical experts who did not diagnose SV in patient i. Then the probability of agreement between the experts in their SV diagnosis of patient i is given by ${p}_{i}:=\frac{1}{m\left(m-1\right)}\left({n}_{i0}\left({n}_{i0}-1\right)+{n}_{i1}\left({n}_{i1}-1\right)\right)$ and ${p}_{\lambda }:=\frac{1}{n}{\sum }_{i=1 }^{n}{p}_{i}$ denotes the mean of this agreement in SV diagnosis. The relative frequency of the diagnosis or non-diagnosis of SV in relation to the total of all clinical examinations is given by ${p}_{1}:= \frac{1}{n\bullet m}{\sum }_{i=1 }^{n}{n}_{i1}$(SV) or ${p}_{0}:= \frac{1}{n\bullet m}{\sum }_{i=1 }^{n}{n}_{i0}$(no SV) and ${p}_{e}:={p}_{0}^{2}+{p}_{1}^{2}$.

By combining an inter-item correlation with an inter-rater correlation, the clinical inspection of SV can be thoroughly assessed. Cronbach's $\alpha$ as a measure of inter-item correlation quantifies how well the selected images represent SV, Fleiss κ as a measure of inter-rater reliability quantifies how well medical professionals agree in their findings on these data.

#### Estimation of the maximum linear SV correlation

The reliability R of a measurement is the proportion of the variance of the true values τ in the total variance of the observed values X: $R:=\frac{{\sigma }_{\tau }^{2}}{{\sigma }_{X}^{2}}$.

We estimate the SV reliability in the clinical inspection experiment by split-half correlation in the parallel measurement model on the set of m = 23 medical experts (Fig. 3).

For this purpose, the m = 23 medical experts were divided into two groups of approximately equal size and the mean values SV_P1_, SV_P2_ of the SV inspection of both groups were calculated for each image. With respect to a fixed image i, we assume that SV_P1_ and SV_P2_ estimate the same true value ${\tau }_{i}$ with the same errors (parallel measurement model). This is plausible because the image is the same and a priori there is no difference in error variance to be assumed between the groups of the two SV_P_ values. The test–retest correlation of the two SV_P_ scores (meaning that first mean value $\mu$_1i_ and then mean value $\mu$_2i_ (see Fig. 3) estimates the true value ${\tau }_{i}$ of image i) is then an estimator for the reliability of the SV inspection. Note, that Cronbach's α is not an estimate of reliability because it measures how accurately the selected images represent SV, rather than of the accuracy of the clinical inspection of SV.

Knowing the reliability R of a measurement X limits the maximum possible linear correlation r with an arbitrary other parameter Y by

$$r\left(X,Y\right):=\frac{cov\left(X,Y\right)}{\sqrt{{\sigma }_{X}^{2}}\sqrt{{\sigma }_{Y}^{2}}}= \frac{cov\left(X,Y\right)}{\frac{\sqrt{{\sigma }_{\tau }^{2}}}{\sqrt{R}}\sqrt{{\sigma }_{Y}^{2}}}=\sqrt{R}\frac{cov\left(X,Y\right)}{\sqrt{{\sigma }_{\tau }^{2}}\sqrt{{\sigma }_{Y}^{2}}}\le \sqrt{R}\cdot 1.$$

#### Qualitative analysis of the RA score 

The analysis of the RA score $RA:=\frac{A}{{L}^{2}}$ (A: Area of visible sublingual veins (px^2^), L: tongue length (px^2^)) was performed with a receiver operating characteristics (ROC) curve against SV_P_ as surrogate parameter for SV. Numerical analysis was performed by calculating the condition numbers ${\varphi }_{A}$, ${\varphi }_{L}$ for the input parameters A, L. For $i = 1,...,n$, the condition number ${\varphi }_{i}$ of a task $f\left(x\right)$ with respect to the i-th argument is defined as:

$${\varphi }_{i}:=\frac{{x}_{i}}{f\left(x\right)}\frac{\partial f}{\partial {x}_{i}}\left(x\right)$$

Condition numbers are a quantitative measure of the dependence of the target parameters on disturbances in the input data. The condition number ${\varphi }_{i}$ is the factor by which the input error of the input parameter i can be amplified maximally, i.e. in the worst case, by the calculation [19].

## Results

### Inspection of SV is a difficult task of clinical examination

**Fig. 4:**
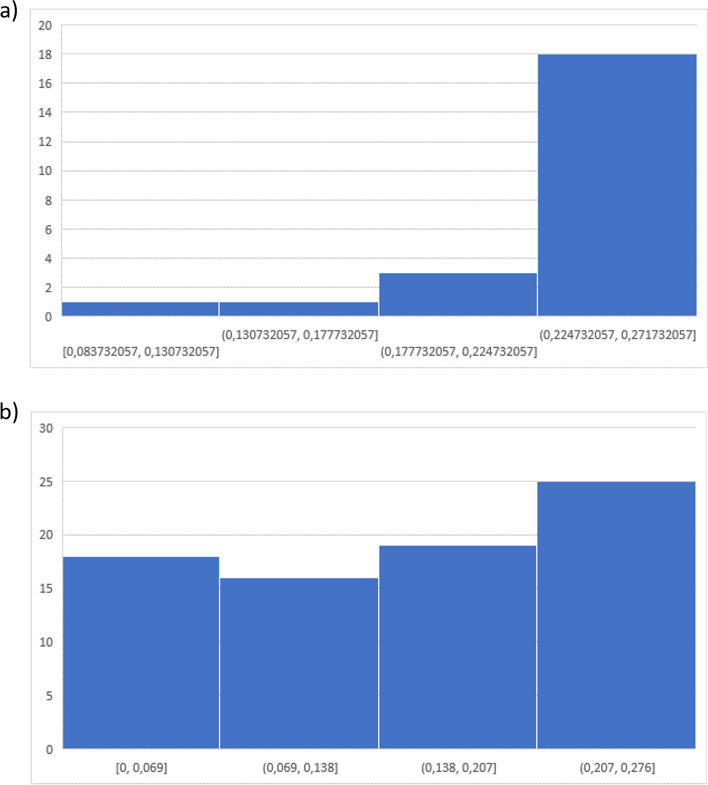
Clinical SV inspection experiment. Visualisation of Cronbachs α: a) Histogram of observers variances (0/1 classification). b) Histogram of Variances of individual images (0/1 classification)

We determined the difficulty of clinical inspection of SV using Cronbach's α = 0.937 and Fleiss' κ = 0.397. The sum of the variance of the individual images ${\sum }_{i=1}^{n}{\sigma }_{{U}_{i}}^{2}$ was comparatively small with respect to the total variance ${\sigma }_{V}^{2}$. The variance of the observers with respect to the images was larger than the sum of the variances of the images across all observers. Cronbach's α thus indicates that the clinical examination of SV was, in principle, possible. The interrater reliability was relatively small with κ = 0.397. Along these lines, inspection of SV was difficult to reproduce and was a difficult task of clinical examination. With Cronbach's α≈ 0.937, the low non-random agreement was due to variance in individual observer ratings rather than variance in individual image ratings (Fig. 4).

### Maximum correlation of SV with an arbitrary other parameter Y

**Fig. 5:**
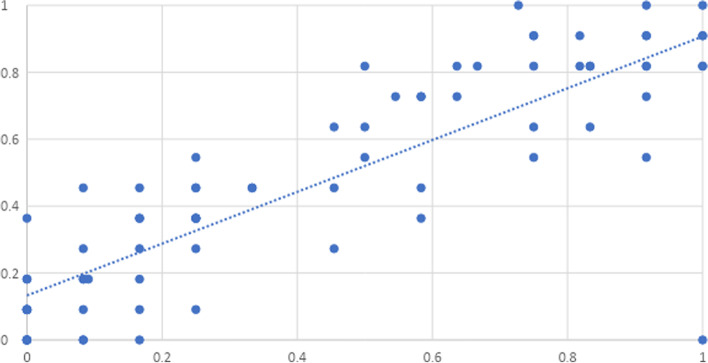
Special test–retest correlation for estimating reliability in the parallel measurement model. The correlation estimates the reliability of the measurement of SV. This limits the maximum possible linear correlation of SV with an arbitrary other parameter Y

To estimate the maximum correlation of SV with an arbitrary other parameter Y, we estimated the reliability R of the SV inspection by a special test–retest correlation (Fig. 5). We thus obtained an inspection reliability for SV of *R* = 0.847. This resulted in a maximum linear correlation of ${r}_{max}$(SV_P_, Y) = 0,920.

### RA score as an objective marker and continuous classification system for SV

**Fig. 6:**
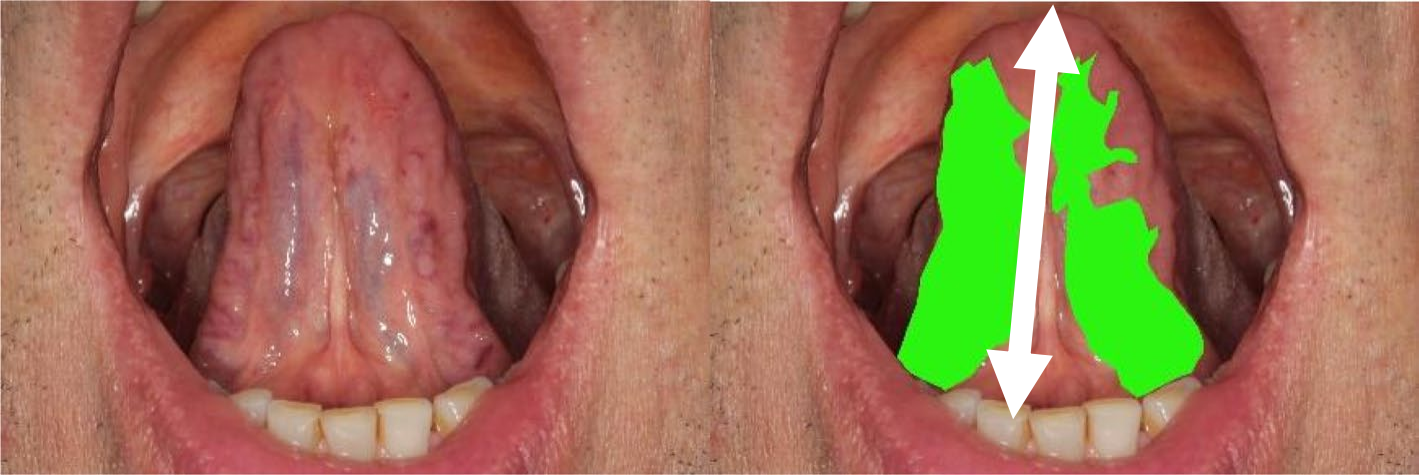
Exemplary representation of the coloured tongue area for calculating the RA score. Written consent obtained from patient. The area of the sublingual veins (*Vv. linguae profundae*) or their dilatation is measured (e.g. by colouring with GIMP) in px^2^ and then normalised to the square of the tongue length (along the *frenulum linguae*): $RA :=\frac{A}{{L}^{2}}$

To overcome the problem of insufficient reliability in clinical inspection, we propose the relative area score (RA) as a new objective measure and continuous classification system for SV. Since absolute tongue areas of visible vein wall dilatation are not well comparable due to different sizes and inspection ratios, tongue length is used as a reliable normalization: The RA score is determined as the area of visible sublingual veins $A$ (pixels(px)^2^) normalized to the square of the tongue length ${L}^{2}$ (px^2^): $RA :=\frac{A}{{L}^{2}}$ (Fig. 6). This score is a non-dimensional measure in the range of values 0 < RA <  < 1. The test–retest reliability of the RA determination is good with a variance of about < 10 × in A and L. In each case, the RA score correlates better with SV_P_ than the individual variables A and L. It thus provides a continuous, and largely viewer-independent scoring system for SV. As the definition of the RA score does not distinguish between the left and right sides of the tongue, a camera position close to the saggital axis is required for a stable calculation in order to reliably capture the entire underside of the tongue.

For the condition numbers of the RA value with respect to A, L we obtain

$${\varphi }_{A } := \frac{A}{RA}\cdot \frac{\partial RA}{\partial A}\left(A,L\right)= \frac{A\cdot {L}^{2}}{A}\cdot \frac{1}{{L}^{2}}=1$$

$${\varphi }_{L} := \frac{L}{RA}\frac{\partial RA}{\partial L}\left(A,L\right)= \frac{{L}^{3}}{A}\left(-2\right)\frac{A}{{L}^{3}}= -2$$

Due to small absolute value of the condition numbers, the RA score is well conditioned. This means that if the input parameters A, L are disturbed, no excessive error amplification is to be expected from the calculation itself. In this sense, the RA score is a useful measure from a numerical point of view.

**Fig. 7:**
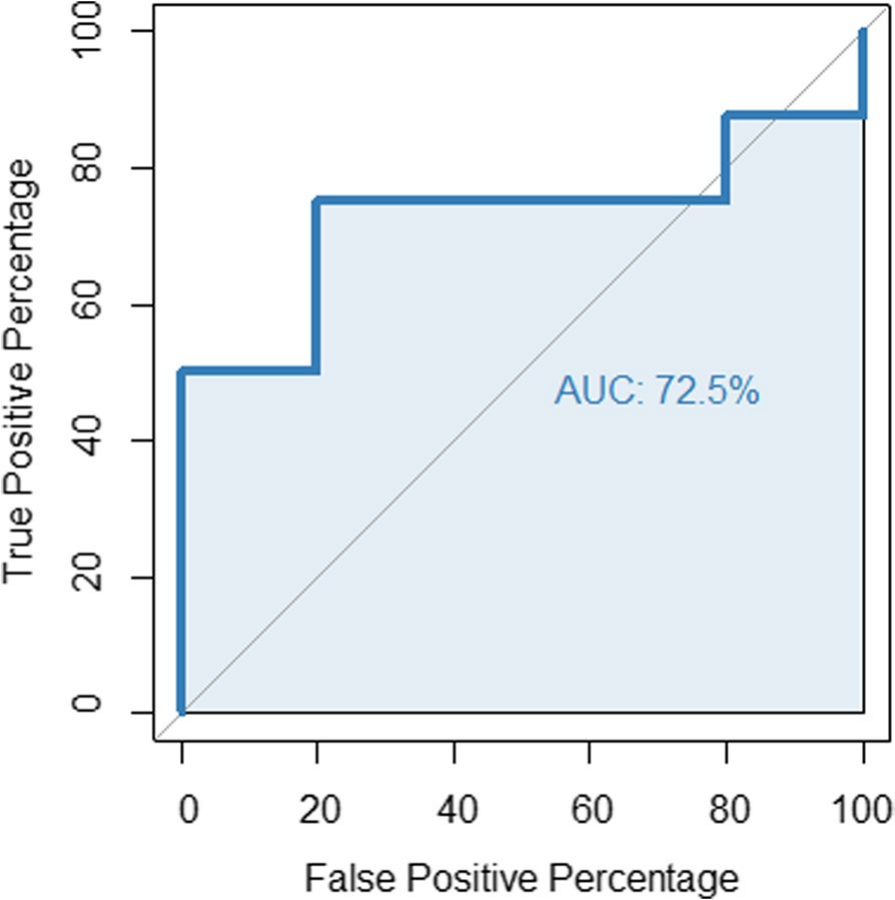
ROC (receiver operating characteristic) curve for the RA score based on SV_P_. The AUC is 72.5%

Figure 7 shows the ROC curve for the RA score based on SV_P_ with AUC = 72.5%. Since other parameters besides the 2D projection of vein wall dilatation are taken into account in the clinical SV inspection, no 100% discriminatory accuracy can be expected.

With an AUC of 72.5%, the RA shows a good discriminatory power regarding SV_P_. Since the RA score only takes into account the 2D projection of vein wall dilatation and not other factors such as topography or colour distribution of the sublingual veins, it can be concluded that the estimation of SV_P_ by medical experts is similarly influenced more by geometric impressions than by others that the RA score cannot measure. Likewise, the RA score can also be used to evaluate automated SV segmentation.

## Discussion

SV are a widely studied area of oral medicine. Many previous studies have diagnosed SV on a binary scale (0/1) with relatively few observers [1, 2, 4–6, 8, 14]. We systematically investigated the influence of the SV inspection reliability for the first time and found that SV inspection is possible in principle, but its inspection reliability is relatively small. SV diagnosis in clinical inspection has greater heterogeneity than previously thought. This finding may be important for the design of future SV studies, as the inclusion of a larger number of experts could better represent the SV inspection and thus increase the reproducibility of the SV inspection.

The SV inspection reliability also limits a priori the maximum possible correlation SV with other clinical parameters. It can be seen that the maximum possible correlation with any other parameter Y can in principle never reach 100% due to the measurement uncertainty of SV. Reliability is therefore an essential quality feature for SV as a predictive marker. The design of future studies on SV as a predictive marker can thus benefit from controlling for inspection reliability.

The limited reliability of SV inspection should also be taken into account when interpreting previous studies. Previous work did not calculate a linear correlation when analysing SV as a predictive marker for other metric parameters (such as arterial blood pressure levels), as this only exists for metric variables. Methodologically, we defined a metric variable by introducing the surrogate parameter SV_P_, which allows a (linear) correlation of the SV inspection with other metric parameters. This approach may also be helpful for further diagnostic studies on SV as a marker for metric outcome parameters.

To increase reliability and objectivity of SV inspection, we propose the RA score. This relates the 2D projection of the sublingual veins A to the square of the tongue length L. The RA score is a largely examiner-independent measure with a high reliability of A and L. In this way, it can be used as a point of comparison and normalisation of different clinical inspection findings in the context of SV diagnostics. The metric range of values maps the continuity of the anatomical variance SV more accurately than a binary 0/1 classification and enables many statistical applications such as (linear) correlation. From an algorithmic point of view, the RA score is well conditioned and could therefore be used in automated SV segmentation [20–22] in addition to clinical SV studies. As there is no gold standard for defining and measuring SV, the evaluation of the RA score can only be a relative one. The ROC curve (Fig. 7) was determined on the SV_P_ as surrogate parameter and shows a relatively good prediction with an AUC = 72%. Regardless of this, the RA score must prove itself as a measure in practice.

A probable limitation of this work is the sample size of *n* = 78 patients in the clinical study. It would be interesting to apply the presented methods for inspection reliability to a larger cohort. By using a frequentist concept of SV (SV_P_), it would be possible to obtain a more precise prevalence estimate of SV and a more stable estimate of the maximum possible correlation ${r}_{max}$ (from the reliability estimate R). In addition, RA score calculation could be automated using machine learning methods, as has already been established for binary (0/1) SV classification [23].

## Conclusions

For the first time, the inspection reliability SV has been systematically investigated. It turned out that SV inspection is possible in principle, but the inspection results were not always stably reproducible across different medical experts. This limits the maximum possible correlation of SV with other parameters, a correlation of 100% does not seem to be achievable a priori. The reliability of SV inspection is important for the quality of SV as a predictive marker. This should be taken into account when interpreting previous studies on SV and has implications for future studies. The RA score could help to objectify the SV examination and thus increase its reliability. To optimise the accuracy of SV diagnosis in clinical practice, we recommend calculating the RA score in systematic examinations of SV rather than using a subjective examiner-dependent assessment as the basis for diagnosis. In this way, the quality of examiners' findings can be compared. In addition, the use of the RA score in clinical practice allows clinicians who do not routinely perform oral examinations to include the diagnosis of SV in their assessments and diagnoses. In this way, the RA score opens up the possibility of including the diagnosis of SV in the diagnosis of other medical specialties outside of oral medicine.

## Data Availability

The data generated in this study are not publicly available due to patient data privacy but are available upon reasonable request from the corresponding author. The raw data supporting the conclusions of this article will be made available by the authors, without undue reservation.

## Abbreviations

Px: Pixels
R: Reliability
ROC: Receiver operating characteristics
SV: Sublingual varices
SV_P_: SV subject prevalence
